# Influence of Zn, Cd, and Cu fractions on enzymatic activity of arable soils

**DOI:** 10.1007/s10661-018-6651-1

**Published:** 2018-04-12

**Authors:** Adam Łukowski, Dorota Dec

**Affiliations:** 1 Department of Technology and Environmental Engineering Systems, Bialystok University of Technology, Wiejska 45A, 15-351 Białystok, Poland; 2 Department of Agri-Food Engineering and Environmental Management, Bialystok University of Technology, Wiejska 45A, 15-351 Białystok, Poland

**Keywords:** Heavy metal, Metal fractionation, BCR method, Enzyme

## Abstract

The aim of this study was to investigate the heavy metal effect on enzymatic activity in acidic soil samples during spring, summer, and autumn. The four metal fractions, acid-soluble and exchangeable (F1), reducible (F2), oxidizable (F3), and residual (F4) using BCR method in soil samples, were evaluated. The highest percentage share of zinc and copper was determined in F4 (45.8, 54.9%, respectively) and cadmium in F3 (45.6%). The enzymatic activity in soil was differentiated in seasons. During spring, the significant relationship was noted between F1/zinc/dehydrogenase, during summer F2/cadmium/phosphatase as well as F4/cadmium/dehydrogenase and autumn F3/zinc/dehydrogenase. Fraction F1/zinc/copper influenced phosphatase activity, whereas F3/Zn increased dehydrogenase and F2/Cd protease activity. The results indicate that the heavy metals affected dehydrogenase activity the most.

## Introduction

The heavy metals are potentially toxic to plants, animals, and humans (He et al. 2013; Hanumanth Kumar and Pramoda Kumari 2015). Toxicity of elements is connected mainly with blocking of enzyme active sites, displacing some cations which are essential for functioning of cell and repossession of their functions. The harmful effects of mineral xenobiotics on plants are related to their mobility (Fang et al. 2012; Szolnoki and Farsang 2013).

Due to anthropogenic activities, such as mining, incineration of wastes, and agricultural practices (i.e., pesticides and sewage sludge application), soils can be contaminated by heavy metals, such as zinc, cadmium, and copper. Phytotoxic concentrations of contaminants can cause limitation of vegetation (Puga et al. 2015).

Zinc is an essential micronutrient for plant development and growth (Kashem et al. 2010) and it acts as cofactor of more than 300 proteins. It is present in all six classes of enzymes (Gupta et al. 2016). Zinc belongs to metals in particular active in soil and thus can be phytotoxic in high concentrations. Its toxicity is dependent on the plant development stage, soil properties, and plant species. Cereals belong to the plants which are most sensitive to zinc excess in the soil. They give a lower yield in such conditions (Baran 2013). Cadmium is a very mobile element in the environment (Łukowski and Wiater 2015a), easily absorbed by roots and transported to shoots. It is uniformly distributed in plant organs (Ciećko et al. 2004; Sękara et al. 2005). Generally, the concentration and accumulation of this metal in plants are positively associated with the soil-soluble Cd fractions (Gao et al. 2011). Availability of Cd to plants is related to pH, soil organic matter, and redox potential (Gao et al. 2011; Brokbartold et al. 2012; Kacálková et al. 2014). Cadmium inhibits activity of photosynthetic enzymes, decreases chlorophyll content, increases membrane conductance, and causes oxidative stress, resulting in inhibition of photosynthesis and growth (Duchovskis et al. 2006). The copper is an essential element for proper growth and development of plants (Łukowski and Wiater 2015b). Copper in plants is functioning as a catalyst in respiration and photosynthesis. It is vital element for the creation of lignin in plant cell walls and in the case of enzymes responsible for protein synthesis. It also influences the disease resistance and reproduction. Copper toxicity can be the cause of deficiency of some nutrients which is connected with plasma membrane consistency and cell wall damage (Matijevic et al. 2014; de Freitas et al. 2015). It is related also to the plant dwarfing and loss of vigor and reduced seed germination as well as root malformation and chlorosis (Elhawat et al. 2015). A direct effect of high Cu concentrations at the cellular level is oxidative stress caused by the increased concentration of reactive oxygen species, such as superoxide anion (O^2−^) or hydrogen peroxide (H_2_O_2_). Excessive Cu can affect the biological activity of soils (Miotto et al. 2014).

The metals when accessing the cell can combine with protein and block functional groups of many enzymes. The heavy metals accumulated in the soil inhibit growth of microorganisms and lead to disturbance in basic physiological functions related mainly to decomposition and transformation of organic matter. Metal ions influence growth rate of organisms, fungal spores, and their enzymatic activity (Badura and Piotrowska-Seget 2000). Microorganisms (bacteria, fungi) possess often accommodative mechanisms which allow to survive and adapt in heavy metal-contaminated environment. The ability to adapt to adverse environmental conditions is connected with metabolic functions, such as specific transport of metal ions with the use of permeases located in cytoplasmic membrane (Binet et al. 2003) as well as synthesis and exudation into environment the chelating agents which are able to bind and transport ions in the environment (Paul et al. 2007). In the case of some bacteria, non-specific metal accumulation by bacterial mucilage and binding by biopolymers from the group of membranes can occur (Ledin 2000).

The biological activity of the soil is evaluated mainly on the basis of the activity of four enzymes: dehydrogenase, phosphatase, urease, and protease (Dec 2014). Often, only dehydrogenase activity is used for this purpose (Kaczyński et al. 2016). The heavy metals inhibit enzymatic and microbiological activity in the soil due to changes in microflora composition and activity of individual enzymes which decreases organic matter decomposition. Negative influence of Zn and Cu on dehydrogenase and urease was stated by Chaperon and Sauve (2007) while Cd inhibits activity of phosphatases and urease (Khan et al. 2010). Lorenz et al. (2006) have reported that long-lasting heavy metal excess adversely affects the microbiological and biochemical activity of soil. Soil contaminated with cadmium in amounts 50 and 250 mg kg^−1^ after 25 years still contained 34 and 134 mg kg^−1^ Cd. The investigation of soil enzymatic activity is useful for assessment of its chemical degradation.

The aim of the studies was determination of zinc, cadmium, and copper fractions and their influence on enzymatic activity (dehydrogenase, protease, alkaline phosphatase, urease) of arable soils cultivated by simplified method.

## Material and methods

### Soil sampling

**Fig. 1:**
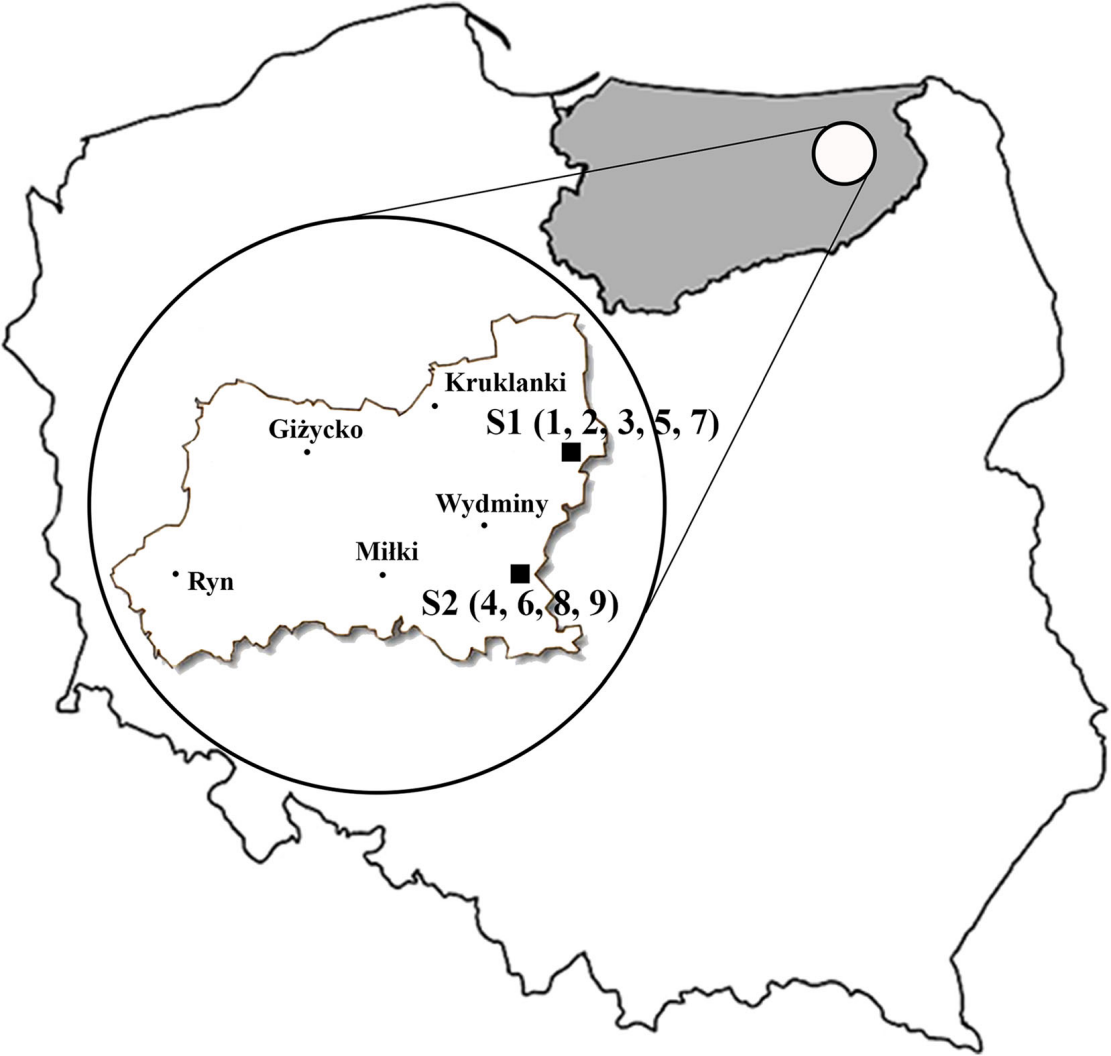
Location of sampling sites. S1—Gawliki Wielkie, S2—Radzie (sample numbers in parenthesis)

The study area is located in north-eastern Poland in the Warmian-Mazurian Voivodeship, Gołdap County, near the Gawliki Wielkie (S1) and Radzie (S2) (Fig. 1). Both localities are part of the Masurian Lake District. Most of the soils in this area have boulder clay origin. Nine soil samples (*Albic Luvisols*) from the agricultural fields covering 150-ha area were collected three times (April, July, and October) during the growing season in 2015, from a depth of 0–25 cm. Each sample (Ahumada et al. 2009; Ahumada et al. 2014; Azouzi et al. 2015; Badura and Piotrowska-Seget 2000; Baran 2013; Binet et al. 2003; Brokbartold et al. 2012; Chaperon and Sauve 2007; Cheng et al. 2011) consisted of six subsamples. Non-ploughing techniques of cultivation are used in this area (the fields have not been ploughed for 5 years). This is novelty in Poland and that is why it is important to investigate the soil from these sites. In the sampling site S1 area, the winter wheat was cultivated. The following fertilization was applied: 287.5 kg N ha^−1^, 75 kg P ha^−1^, 30 kg S ha^−1^, and 120 kg CaO ha^−1^. In the S2 site, where the broad bean grew, the following fertilization was used: 45 kg N ha^−1^, 75 kg P ha^−1^, 8 kg S ha^−1^, and 120 kg CaO ha^−1^.

### Analytical techniques

In soil samples, the pH in 1 mol dm^−3^ KCl (PN-ISO 10390 1997), organic carbon (PN-ISO 14235 1998), and total nitrogen content using Kjeldahl method (PN-ISO 11261 2002) were determined. The soil texture was determined by sieve method with the aerometric method according to the polish regulations: PN-R-04032 and PN-R-04033 (1998) (sand diameter was 2–0.05 mm, silt diameter was 0.05–0.002 mm, clay diameter was ˂ 0.002). The total content of zinc, cadmium and copper was determined on aqua regia extracts (PN-ISO 11466 2002) followed by an analysis of atomic absorption spectrometry (AAS). The content of metals in fractions was determined by means of graphite furnace atomic absorption spectrometry (GFAAS) method. The percentage of individual fractions in total content of each element was calculated. Recovery was calculated as follows: recovery (%) = (sum of the four fractions ∕ total content) × 100.

**Fig. 2:**
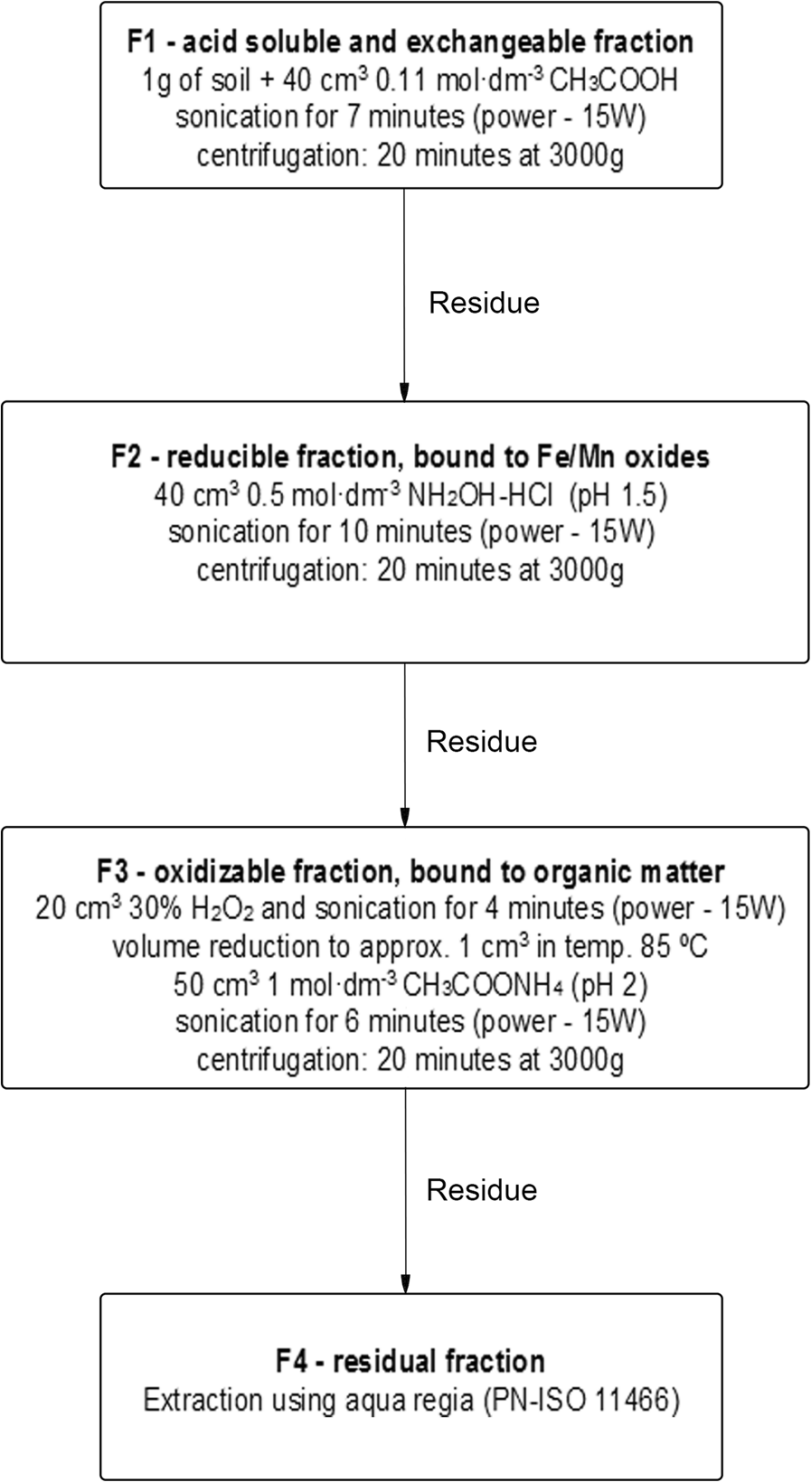
The sequential extraction scheme for the heavy metal partitioning

Modified BCR (Community Bureau of Reference) method with usage of ultrasonic probe Sonics VCX 130 (Leśniewska et al. 2014) was used to evaluate fractional composition of Zn, Cd, and Cu in soil samples. Extraction included four stages (Fig. 2).

### Enzyme activities

Dehydrogenase activity was determined by means of spectrophotometry method (Thalmann 1968). The air-dried soil sample (6 g), after passing through a 2-mm sieve, was added to a 50-cm^3^ conical flask. Then, 60 mg CaCO_3_, 4 cm^3^ of distilled water, and 1cm^3^ of 1% TTC (triphenylotetrazole chloride) were introduced. The content of the flask was mixed and incubated for 24 h at 30 °C. After incubation, 25 cm^3^ of ethanol was added and the flask was stored for 1 h in dark place. The suspension was filtrated through the filter paper (Whatman No. 5). The absorbance was measured at 485 nm. Alkaline phosphatase was assayed by the method of Tabatabai and Bremner (1969). One gram of air-dried and screened soil, 4 cm^3^ of modified universal buffer adjusted to pH = 6.5 (Gomori 1955), 0.25 cm^3^ of toluene, and 1 cm^3^ of 0.115 mol dm^−3^ disodium p-nitrophenylphosphate tetrahydrate were mixed in a 50-cm^3^ conical flask. The flasks were placed in a water bath (37 °C) for 1 h. Then, 1 cm^3^ of 0.5 mol dm^−3^ calcium chloride and 4 cm^3^ of 0.5 mol dm^−3^ sodium hydroxide were added to each flask and mixed. Finally, the soil suspension was filtered through the filter paper (Whatman No. 5). The absorbance of the filtrate was measured in a spectrophotometer at 400 nm. The amount of p-nitrophenol in the samples was then calculated against the standard. Urease activity was measured spectrophotometrically by the method of Hoffmann and Teicher (1961). Fifty gram of air-dried and screened soil, 1 cm^3^ of toluene, 10 cm^3^ of 10% urea solution, and 20 cm^3^ of sodium citrate-citric acid buffer (pH 6–7) were mixed in a 50-cm^3^ volumetric flask. The soil suspension was well shaken and incubated for 3 h at 37 °C. The contents of each flask were then diluted to 50 cm^3^ with distilled water, shaken well, and filtered through Whatman filter paper No. 5. Then, 1 cm^3^ of the clear filtrate was taken and diluted with 9 cm^3^ of distilled water, 4 cm^3^ of freshly prepared sodium phenate, and 3 cm^3^ sodium hypochlorite solution. Next, the color developed was measured with spectrophotometer, using a wave length of 630 nm*.* Protease activity was assayed according to the method of Macura and Vágnerová (1969). One gram of air-dried and screened soil, 0.4 cm^3^ of toluene, and 2 cm^3^ of 1% azo-casein solution (pH 8.3) were mixed in a 10-cm^3^ tube. The contents were placed for 24 h at 37 °C in a water bath. One hour before the end of incubation, 3 cm^3^ of 1% sodium hydrogen carbonate and after incubation 3.5 cm^3^ of 5% trichloroacetic acid were added. Next, soil suspension was filtered through Whatman filter paper No. 5. Then, 5 cm^3^ of the clear filtrate was taken, diluted with 5 cm^3^ of 0.5 mol dm^−3^ sodium hydroxide and its absorbance was measured in a spectrophotometer at 430 nm.

### Statistical analysis

Correlation between studied parameters was calculated by using Pearson’s correlation factor for *P* ≤ 0.05 in Statistica 12.5 software. All data (except sand, silt, and clay content) were subjected to one-way analysis of variance (ANOVA) using Statistica 13.1 software. The least significant difference test was used to detect significant differences among the means of the soil enzyme activities, soil physicochemical properties, and fractions of Zn, Cd, and Cu (*P* < 0.05).

## Results

Textural classes of studied soils varied among clay loam, loam, and sandy loam (Table 1). Soil samples were slightly acidic and acidic according to pH values. They were characterized in the majority by high organic carbon content. Total nitrogen content was characteristic for mineral soils. Total content of Zn, Cd, and Cu was typical for non-contaminated arable soils.

**Table 1:** Characteristics of studied soils (mean ± SD)

Sampling site	Protease (mg azo-casein g^−1^ h^−1^)	Phosphatase (mM pNP g^−1^ h^−1^)	Dehydrogenase (μg TPF g^−1^ DM 24 h^−1^)	Urease (μg N g^−1^ DM h^−1^)	pH	Organic C (%)	N (%)	Sand (%)	Silt (%)	Clay (%)	Zn (mg kg^−1^)	Cd (μg kg^−1^)	Cu (mg kg^−1^)
1	24.26 ± 2.07	0.89 ± 0.24	0.11 ± 0.05	7.36 ± 0.83	6.5 ± 0.28	1.87 ± 1.68	0.31 ± 0.02	20.9 ± 14.5	25.0 ± 9.0	27.6 ± 4.1	54.2 ± 23.6	77.1 ± 22.6	9.3 ± 4.9
2	22.27 ± 1.91	1.66 ± 0.17	0.11 ± 0.01	8.43 ± 0.41	5.7 ± 0.65	2.05 ± 0.35	0.19 ± 0.01	21.9 ± 1.1	15.6 ± 4.9	27.4 ± 2.0	85.2 ± 6.2	105.9 ± 18.1	16.1 ± 0.9
3	21.04 ± 2.05	0.96 ± 0.29	0.22 ± 0.12	6.38 ± 0.92	4.9 ± 0.67	2.02 ± 0.21	0.27 ± 0.03	22.4 ± 6.6	24.6 ± 4.7	30.7 ± 5.2	62.3 ± 14.0	61.0 ± 21.4	12.2 ± 3.1
4	24.27 ± 0.72	0.93 ± 0.21	0.34 ± 0.18	5.09 ± 1.28	6.5 ± 0.91	2.37 ± 1.52	0.32 ± 0.13	27.2 ± 9.1	29.9 ± 1.9	7.6 ± 0.9	41.5 ± 12.2	54.4 ± 11.4	10.7 ± 3.5
5	22.40 ± 1.64	1.66 ± 0.88	0.23 ± 0.14	5.65 ± 0.52	5.7 ± 0.69	2.25 ± 0.30	0.31 ± 0.15	23.4 ± 0.7	22.7 ± 5.2	31.4 ± 3.5	56.0 ± 26.4	51.4 ± 11.3	10.4 ± 5.8
6	21.08 ± 1.91	0.72 ± 0.27	0.18 ± 0.11	6.61 ± 1.76	5.2 ± 0.89	1.19 ± 0.33	0.22 ± 0.05	39.0 ± 11.6	32.0 ± 4.6	8.2 ± 1.5	48.1 ± 28.2	58.2 ± 3.4	7.6 ± 4.8
7	26.68 ± 5.56	1.41 ± 0.80	0.19 ± 0.15	7.43 ± 1.23	5.7 ± 0.14	3.36 ± 2.68	0.23 ± 0.04	22.1 ± 1.8	30.1 ± 3.9	29.6 ± 3.8	40.4 ± 8.3	36.8 ± 4.6	8.4 ± 6.9
8	22.43 ± 1.55	1.07 ± 0.61	0.16 ± 0.15	6.40 ± 0.68	5.9 ± 1.22	1.48 ± 0.35	0.28 ± 0.07	36.2 ± 24.2	31.5 ± 2.4	7.3 ± 2.1	36.8 ± 9.8	55.9 ± 5.3	7.0 ± 3.2
9	24.39 ± 1.78	0.38 ± 0.05	0.04 ± 0.02	6.18 ± 1.12	5.1 ± 1.79	1.12 ± 0.27	0.21 ± 0.02	56.1 ± 8.3	16.5 ± 1.3	8.3 ± 1.4	28.5 ± 3.9	44.9 ± 6.9	5.4 ± 1.1

In our study, most of zinc was noted in fraction F4 (45.8% of total content on average), and the least in fraction F1 (12.8% on average) (Table 2). Fractions bound to Fe/Mn oxides and organic matter have contained 24.0 and 22.5% of total zinc on average, respectively. In the case of cadmium, the highest percentage share was noted in fraction F3 (45.6% of total content on average) and the lowest in fraction F4 (17.5% on average). In fractions F1 and F2, on average was 38 and 45.2%, respectively. The least percentage share of Cu was stated in fraction F1 (7.8% on average). Fraction F2 constituted 20.1%, fraction F3 27.5%, and fraction F4 54.9% of total copper on average. Average recovery amounted from 93 to 111, 140 to 152, and 94 to 131% for zinc, cadmium, and copper respectively.

**Table 2:** Fractions of Zn, Cd, and Cu (%) in studied soils (mean ± SD) and recovery (%)

Sampling site	Zn					Cd					Cu				
	F1	F2	F3	F4	Recovery	F1	F2	F3	F4	Recovery	F1	F2	F3	F4	Recovery
1	10.1 ± 7.9	28.3 ± 16.8	20.2 ± 7.6	52.4 ± 15.2	111	41.5 ± 3.3	47.3 ± 2.3	38.8 ± 9.2	12.0 ± 9.7	140	4.6 ± 0.5	23.9 ± 18.3	21.9 ± 18.4	48.0 ± 21.3	112
2	4.3 ± 3.6	17.4 ± 9.2	13.4 ± 10.5	58.1 ± 9.5	93	31.6 ± 8.6	45.1 ± 2.0	57.0 ± 11.8	11.8 ± 2.8	145	2.5 ± 1.4	13.7 ± 7.8	4.7 ± 3.2	59.9 ± 42.6	105
3	6.7 ± 1.3	17.0 ± 8.9	36.6 ± 38.7	45.2 ± 19.9	106	46.8 ± 7.2	48.2 ± 3.9	43.2 ± 18.5	14.2 ± 8.1	152	4.3 ± 2.1	16.9 ± 8.7	36.1 ± 42.4	40.9 ± 40.4	115
4	8.4 ± 4.3	35.4 ± 21.1	16.5 ± 9.4	50.1 ± 12.2	110	37.5 ± 1.6	42.2 ± 4.5	47.8 ± 14.3	21.0 ± 6.7	148	5.9 ± 4.0	11.6 ± 8.1	19.8 ± 19.2	38.0 ± 31.4	94
5	7.2 ± 4.4	23.2 ± 16.5	14.1 ± 7.1	56.3 ± 13.2	101	38.7 ± 5.2	40.1 ± 2.8	50.1 ± 12.5	21.1 ± 10.0	150	8.6 ± 5.9	25.3 ± 23.4	24.5 ± 5.3	57.6 ± 14.4	107
6	15.0 ± 6.0	21.7 ± 17.8	19.5 ± 5.2	42.6 ± 20.8	99	42.2 ± 11.9	33.6 ± 4.2	59.6 ± 10.4	16.8 ± 11.6	152	14.2 ± 2.3	14.1 ± 12.6	46.3 ± 33.1	46.8 ± 28.3	131
7	11.1 ± 5.7	27.6 ± 10.2	26.1 ± 5.4	46.5 ± 11.3	111	32.4 ± 0.3	42.2 ± 4.3	41.2 ± 7.4	26.3 ± 6.8	142	7.6 ± 3.3	22.0 ± 15.8	43.6 ± 5.9	81.6 ± 71.5	108
8	22.0 ± 6.7	25.6 ± 14.8	25.3 ± 9.8	34.9 ± 20.3	108	33.8 ± 2.0	44.3 ± 5.7	44.9 ± 11.8	17.9 ± 11.6	141	9.7 ± 4.7	23.1 ± 11.1	20.4 ± 9.0	81.2 ± 21.8	127
9	30.0 ± 10.0	19.5 ± 10.0	30.4 ± 12.4	26.5 ± 13.5	106	37.8 ± 7.1	64.2 ± 13.6	28.0 ± 20.2	16.8 ± 9.8	147	12.8 ± 2.0	29.9 ± 15.7	30.1 ± 15.1	40.0 ± 5.8	114

*F1*, acid-soluble and exchangeable fraction; *F2*, reducible fraction; *F3*, oxidizable fraction; *F4*, residual fraction

In the case of cadmium, the negative correlation was observed between fraction F2 and pH (*r* = − 0.486) as well as silt (*r* = − 0.490) (Table 3). Its fraction F3 correlated positively with pH (*r* = 0.453). Significant correlation between fraction F1 of zinc and organic carbon (*r* = − 0.421), clay content (*r* = − 0.531), and sand (*r* = 0.670) was stated. Fraction F4 of Zn correlated negatively with sand (*r* = − 0.617). The dependences between fraction F1 of Cu and characteristics of studied soils were similar like in the case of zinc. Correlation coefficients between exchangeable fraction and organic carbon, sand, and clay were as follows: − 0.401, 0.567 and − 0.437. Positive correlation between fraction F2 and sand (*r* = 0.392) was also noted.

**Table 3:** Correlation coefficients between characteristics of studied soils and fractions of Cd, Zn, and Cu

	Cd				Zn				Cu			
	F1	F2	F3	F4	F1	F2	F3	F4	F1	F2	F3	F4
pH	− 0.167	− 0.486*	0.453*	− 0.216	− 0.296	0.371	0.042	− 0.130	− 0.036	0.230	0.103	− 0.037
Organic C	− 0.083	− 0.168	0.059	0.145	− 0.421*	0.097	0.041	0.005	− 0.401*	− 0.268	0.145	− 0.276
Sand	− 0.077	0.160	0.035	− 0.158	0.670*	0.180	0.161	− 0.617*	0.567*	0.392*	0.102	− 0.142
Silt	0.053	− 0.490*	0.268	0.008	− 0.046	0.232	0.248	− 0.283	0.128	− 0.067	0.353	− 0.245
Clay	− 0.001	− 0.089	0.120	− 0.105	− 0.531*	0.001	− 0.025	0.305	− 0.437*	0.108	− 0.037	0.013

*F1*, acid-soluble and exchangeable fraction; *F2*, reducible fraction; *F3*, oxidizable fraction; *F4*, residual fraction

*P < 0.05

Significant correlations between enzyme activities and characteristics of studied soils were not observed (Table 4). The level of enzyme activity varied in a wide range and for dehydrogenase amounted 0.02 to 0.60 μg TPF g^−1^ DM 24 h^−1^, for alkaline phosphatase 0.32–2.88 mM pNP g^−1^ h^−1^, for protease 18.29–34.15 mg azo-casein g^−1^ h^−1^, and for urease 4.12–8.90 μg N g^−1^ DM h^−1^. The average enzyme activity is presented in Table 1.

**Table 4:** Correlations between enzyme activities and characteristics of studied soils

	pH	Organic C	N	Sand	Silt	Clay
Protease	0.253	0.139	0.001	− 0.124	0.047	− 0.030
Phosphatase	− 0.204	0.035	− 0.044	− 0.306	− 0.149	0.200
Dehydrogenase	− 0.240	− 0.299	0.290	− 0.061	0.038	− 0.154
Urease	0.070	0.022	− 0.283	− 0.341	− 0.168	0.266

*P < 0.05

There were significant correlations in soil samples collected in all three sampling dates. Acid-soluble and exchangeable fraction of Zn positively correlated with dehydrogenase (*r* = 0.799) in samples which were taken during April (Table 5). In samples collected in July, the cadmium fraction bound to Fe/Mn oxides influenced significantly the phosphatase (*r* = 0.801) and residual fraction positively influenced dehydrogenase (*r* = 0.723). The most of significant dependences was observed in soil samples from October. In the case of cadmium, the positive correlation (*r* = 0.731) between F2 fraction and protease activity was noted. Fraction F1 of zinc negatively correlated (*r* = − 0.722) with phosphatase and fraction F3 positively (*r* = 0.776) with dehydrogenase. Fraction F1 of copper correlated negatively (*r* = − 0.790) with phosphatase.

**Table 5:** Correlation coefficients between pH, organic carbon, enzyme activities, and fractions of Cd, Zn, and Cu in three sampling dates (*n* = 27)

	Cd				Zn				Cu			
	F1	F2	F3	F4	F1	F2	F3	F4	F1	F2	F3	F4
	Soil sampling in April											
pH	− 0.445	− 0.746*	0.602	− 0.048	− 0.628	0.373	0.172	0.587	− 0.311	− 0.448	0.234	0.080
Organic C	− 0.297	− 0.304	0.103	0.382	− 0.351	0.853*	0.755*	− 0.239	− 0.479	− 0.500	0.200	− 0.659
Protease	0.025	0.036	− 0.279	0.079	− 0.088	0.503	0.308	− 0.253	− 0.410	0.127	− 0.161	− 0.048
Phosphatase	− 0.251	− 0.486	0.401	0.151	− 0.410	0.585	0.523	− 0.105	− 0.425	− 0.381	0.070	− 0.506
Dehydrogenase	0.169	0.545	− 0.551	0.375	0.799*	− 0.394	− 0.007	− 0.469	0.654	0.142	− 0.002	− 0.132
Urease	− 0.138	− 0.254	0.330	− 0.300	− 0.212	0.071	0.058	0.321	− 0.147	0.014	0.431	− 0.157
	Soil sampling in July											
pH	0.128	− 0.727*	0.629	0.117	− 0.532	0.683*	− 0.590	0.510	− 0.009	0.094	− 0.011	0.160
Organic C	− 0.093	− 0.433	0.671*	− 0.018	− 0.783*	− 0.115	− 0.346	0.648	− 0.461	− 0.449	− 0.323	0.822*
Protease	0.048	− 0.342	0.261	0.243	− 0.134	0.572	− 0.316	0.091	0.025	0.123	− 0.054	− 0.228
Phosphatase	− 0.503	0.801*	− 0.527	− 0.073	0.457	− 0.485	0.587	− 0.287	− 0.109	0.073	− 0.193	− 0.285
Dehydrogenase	0.068	− 0.539	0.297	0.723*	− 0.364	0.439	− 0.393	0.013	0.157	− 0.067	0.292	− 0.210
Urease	− 0.113	− 0.412	0.107	0.018	− 0.098	− 0.014	0.397	− 0.003	− 0.017	− 0.119	0.449	0.324
	Soil sampling in October											
pH	− 0.257	0.509	− 0.386	− 0.194	0.095	− 0.124	− 0.042	− 0.308	0.062	0.033	− 0.217	0.212
Organic C	0.480	0.325	− 0.347	0.045	− 0.568	0.171	− 0.096	0.221	− 0.557	− 0.831*	− 0.052	− 0.223
Protease	− 0.473	0.731*	− 0.507	0.149	− 0.098	− 0.214	− 0.088	0.514	0.062	0.366	0.000	− 0.439
Phosphatase	− 0.112	− 0.150	− 0.071	0.316	− 0.722*	− 0.165	− 0.458	0.639	− 0.790*	− 0.645	− 0.598	0.326
Dehydrogenase	0.146	− 0.112	0.491	− 0.514	− 0.246	− 0.346	0.776*	− 0.306	− 0.286	− 0.068	0.554	− 0.130
Urease	− 0.362	0.395	0.264	− 0.530	− 0.436	− 0.277	0.092	0.119	− 0.429	− 0.155	− 0.108	0.276

*F1*, acid-soluble and exchangeable fraction; *F2*, reducible fraction; *F3*, oxidizable fraction; *F4*, residual fraction

*P < 0.05

Percentage share of zinc F1 fraction was not significantly differentiated between sampling dates (Table 6). In the case of other fractions, the significant differences occurred. Similar dependencies were stated for copper fractions. For cadmium, significant differences were noted in the case of fractions F3 and F4. Physicochemical properties of soils were generally not differentiated by sampling dates; only nitrogen content in 2 months was significantly different. Among enzymes, protease and dehydrogenase activities were significantly differentiated.

**Table 6:** Effect of sampling date on soil enzyme activities, soil physicochemical properties, and fractions of Zn, Cd, and Cu

		Sampling date		
		April	July	October
Protease (mg azo-casein g^−1^ h^−1^)		24.35 ± 0.93b	20.92 ± 1.88a	24.08 ± 4.36ab
Phosphatase (mM pNP g^−1^ h^−1^)		1.50 ± 0.78a	1.18 ± 0.53a	0.78 ± 0.45a
Dehydrogenase (μg TPF g^−1^ DM 24 h^−1^)		0.33 ± 0.17b	0.18 ± 0.14ab	0.08 ± 0.07a
Urease (μg N g^−1^ DM h^−1^)		6.96 ± 1.42a	6.45 ± 1.67a	6.43 ± 1.32a
pH		5.29 ± 0.71a	6.01 ± 0.89a	6.17 ± 1.18a
Organic C (%)		2.00 ± 2.05a	2.13 ± 1.00a	3.70 ± 4.32a
N (%)		0.28 ± 0.05ab	0.31 ± 0.12b	0.19 ± 0.04a
Zn (%)	F1	11.73 ± 7.03	15.84 ± 12.39	10.68 ± 10.56
	F2	8.25 ± 4.38a	36.25 ± 13.93b	27.43 ± 12.02b
	F3	12.05 ± 3.05a	21.67 ± 12.19ab	33.64 ± 23.57b
	F4	60.39 ± 8.10a	47.87 ± 16.69a	29.25 ± 15.34b
Cd (%)	F1	41.36 ± 8.15	35.74 ± 10.58	36.99 ± 3.54
	F2	47.60 ± 8.92	45.28 ± 14.38	42.82 ± 4.84
	F3	29.05 ± 11.99b	53.58 ± 14.82a	54.27 ± 7.94a
	F4	28.17 ± 5.94b	9.35 ± 6.97a	15.07 ± 6.63a
Cu (%)	F1	8.28 ± 4.32	6.75 ± 5.41	8.38 ± 5.83
	F2	4.42 ± 3.26b	29.23 ± 16.53a	26.51 ± 10.65a
	F3	25.43 ± 24.59ab	11.64 ± 12.41a	45.41 ± 24.47b
	F4	58.17 ± 16.27a	80.96 ± 19.19a	32.51 ± 23.13b

Values are mean ± SD. Different letters in the same row indicate significant difference (*P* < 0.05)

*F1*, acid-soluble and exchangeable fraction; *F2*, reducible fraction; *F3*, oxidizable fraction; *F4*, residual fraction

## Discussion

Most of studies regarding the effect of metals on enzyme activity are concentrating on total form of metal. Total metal concentrations are poor indicators of toxicity in soils, since large metal fractions are present in biologically unavailable forms (Lazzaro et al. 2006). Determination of bioavailable metals requires evaluation of their soluble and potentially soluble fractions which can be done by usage of sequential extraction.

Zeiner et al. (2013) during study of soils from orchard in residual fraction have stated 56.5% and in exchangeable fraction 0.896% of total zinc. Similar dependence was noted in our study. Cheng et al. (2011) also reported that the lowest amount of Zn (21% of total content) in garden soils has constituted acid-soluble and exchangeable fraction. This fraction is considered as phytoavailable fraction of total metal (Ahumada et al. 2009). Li et al. (2016) in studies of soil contaminated due to mining activities have stated that the highest cadmium amounts, unlike in our study, constituted exchangeable fraction (52.2%). Cui et al. (2013) in soil from heavy metal-contaminated agricultural field found the highest percentage share of this metal in residual fraction (46.7%) and the lowest in organic matter fraction (0.9%). The most of copper was in fraction F4 (54.9% on average). It means that Cu is mainly bound to the mineral structure of soil matrix (Sungur et al. 2016). It is the result similar to that which was noted by abovementioned Zeiner et al. (2013)—50.9% and Ahumada et al. (2014) in studies of agricultural soils—55.8% on average in control soil without biosolids. The content of metal in this fraction indicates environmental pollution. It means that the higher the quantity of metal present in residual fraction, the lower the degree of pollution (Szolnoki and Farsang 2013). The percentage share of Cu which was stated in fraction F1 (7.8% on average) is consistent with results of Szolnoki and Farsang (2013). Authors reported that in soils from urban vegetable gardens in exchangeable fraction, Cu on average was less than 3%. According to study results, fraction F2 constituted 20.1% and fraction F3 27.5% of total copper on average.

Recoveries (Table 2) indicated satisfactory accuracy for all the elements, especially for the zinc (93–111%). Non-quantitative recoveries in BCR method are related to losses (Lu et al. 2009), lack of selectivity of reagents (Gleyzes et al. 2002), contamination (Fernández et al. 2004), and sources of errors influencing analytical results which are not identified so far (Leśniewska et al. 2016).

Significant changes of metal content in fractions between sampling dates were probably related to redistribution of elements. In the case of zinc, there was a shift from fractions F4 to F2 and F3. Part of the copper shifted from fractions F3 to F2 and from residual fraction to F3. Redistribution of cadmium was connected with shifting from fractions F4 to F3.

Mobility and availability of different metal forms are mostly ordered as follows: acid-soluble > bound to Fe/Mn oxides > bound to organic matter > residual forms (Zimmerman and Weindorf 2010). The initial three fractions are commonly considered the mobile fractions and represent the extractability of the elements (Sungur et al. 2016). In our study, cadmium was characterized by the highest extractability, while copper the lowest.

Heavy metals inhibit the enzymatic activity in soils (Wang et al. 2009). They modify the microbial communities, thus synthesis of enzymes. Metals block also the enzyme-binding sites by combining with the active protein groups of enzymes (Cui et al. 2013). Positive correlation between urease and residual fraction of copper (*r* = 0.69, *P* ≤ 0.01) was stated by Cui et al. (2013) in metal-contaminated agricultural soil. Authors reported also negative correlation between urease and residual fraction of cadmium (*r* = − 0.47, *P* ≤ 0.05). They have noted opposite dependency between urease and reducible Cd fraction (*r* = 0.52, *P* ≤ 0.05). Some authors found relationship between physicochemical properties of soil and enzyme activity, for example Kunito et al. (2001) in the case of sludge-amended soils. The authors observed positive significant relationships between alkaline phosphatase, dehydrogenase, protease, urease, and pH as well as organic carbon. The soil pH influences the composition of microbial communities because different strains of microorganisms exist at various reactions (Fernández-Calviño et al. 2010). According to Lee et al. (2002), dehydrogenase activity correlates significantly and positively with organic matter content (*r* = 0.89, *P* ≤ 0.01), but negatively with pH (*r* = − 0.92, *P* ≤ 0.001). Fernández-Calviño et al. (2010) reported that dehydrogenase activity showed significantly positive correlations with vineyard soil pH (*r* = 0.673, *P* < 0.01 for pH_water_) and clay content (*r* = 0.213, *P* < 0.05). They have stated also that the urease activity was negatively related to pH (*r* = − 0.342, *P* < 0.05 for pH_water_), whereas phosphatase activity was positively correlated with organic carbon content (*r* = 0.553, *P* < 0.01) and silt content (*r* = 0.246, *P* < 0.01), while negatively correlated with sand content (*r* = − 0.224, *P* < 0.01).

The negative correlation between fraction F2 Cd and pH in the first two dates and the lack of correlation at the last date can be explained by increase of soil pH due to application of calcium and phosphorus fertilizer to the winter crops. Despite soil pH did not have much effect on adsorption capacity of the oxides (Azouzi et al. 2015) in the case of zinc, correlation between fraction F2 and pH was also found. Strong positive correlation between fraction F2 Zn and organic matter was probably related to the humic material adsorbed on oxides which generally increases metal adsorption at low pH (average pH in April was the lowest among all the sampling dates) (McBride et al. 1997). Surprisingly, there is no significant correlation between fraction F3 Cu and organic matter, since copper has high affinity towards humic substances. The reason for this is unclear. It could be connected with the low percentage share of copper in fraction F3 (11.6% on average) in soil samples from July.

Soil enzyme activities are variable during the year. The activity is usually higher in summer than in winter (Serra-Wittling et al. 1995). Our results showed that all enzymes were most active in spring. Statistically significant differences obtained for protease and dehydrogenase activities in the samplings were probably due to the very different climatic conditions.

## Conclusions

Zinc and copper were bound strongly to the soil minerals, while cadmium to organic matter. The zinc content (mean values) in particular fractions can be arranged quantitatively in a sequence: F4 (45.8%) > F2 (24%) > F3 (22.5%) > F1 (12.8%), in the case of cadmium: F3 (45.6%) > F2 (45.2%) > F1 (38.0%) > F4 (17.5%), and in the case of copper: F4 (54.9%) > F3 (27.5%) > F2 (20.1%) > F1 (7.8%). Assuming the cumulative content of metal in mobile fractions (F1 + F2 + F3) as the solubility criterion, it can be stated that cadmium was the most soluble and copper the least soluble. Cadmium was also the most bioavailable due to the highest content in the most mobile fraction (F1) among studied metals.

In our study, the differentiated influence of metal fractions on soil enzymatic activity in individual sampling dates was observed. During spring, the significant relationship between acid-soluble and exchangeable fraction of zinc and dehydrogenase was stated. During summer, when the soil moisture was very low, the significant relations between reducible fraction and phosphatase as well as between residual fraction and dehydrogenase in the case of cadmium were noted. Such dependence also occurred between oxidizable fraction of zinc and dehydrogenase in October. During autumn, the acid-soluble and exchangeable fraction of zinc and copper caused a drop in phosphatase activity, whereas fraction of Zn bound to organic matter influenced an increase in dehydrogenase activity, thus activity of microorganisms. Protease activity was influenced by F2 fraction of Cd, probably due to utilization of oxygen from Fe/Mn oxides by microorganisms. Protease and dehydrogenase activities were significantly differentiated between sampling dates. Such dependence was also stated in the case of most metal fractions.
